# Current Research in Fermented Foods: Bridging Tradition and Science

**DOI:** 10.1016/j.advnut.2025.100554

**Published:** 2025-11-11

**Authors:** Kara Sampsell, Camila Schultz Marcolla, Samantha Tapping, Yi Fan, Carla L Sánchez-Lafuente, Benjamin P Willing, Raylene A Reimer, Jeremy P Burton

**Affiliations:** 1 Faculty of Kinesiology, University of Calgary, Calgary, Alberta, Canada; 2 Department of Agricultural, Food and Nutritional Science, University of Alberta, Edmonton, Alberta, Canada; 3 Department of Microbiology & Immunology, Western University, London, Ontario, Canada; 4 Department of Biochemistry and Molecular Biology, Cumming School of Medicine, University of Calgary, Calgary, Alberta, Canada; 5 Division of Urology, Department of Surgery, The University of Western Ontario, London, Canada; 6 Lawson Research Institute, London, Ontario, Canada

**Keywords:** fermented foods, gut microbiota, metabolic health, cardiovascular disease, randomized controlled trials, functional foods, microbial bioactives, nutrient bioavailability, noncommunicable diseases, dietary interventions

## Abstract

Fermented foods represent a diverse category of products shaped by microbial metabolism, offering distinctive sensory qualities and potential health benefits. Although prior reviews have explored the nutritional and microbial aspects of fermented foods or focused on specific health outcomes and mechanisms of action, few recent narrative reviews have integrated clinical and epidemiologic evidence across diverse health domains. This review addresses that gap by critically evaluating observational and interventional studies linking fermented food consumption with metabolic, cardiovascular, oncologic, and neuropsychological outcomes, while summarizing associated biomarkers that may underpin these effects. Emphasis is placed on clinical studies of fermented foods containing live microbes. Through mapping current evidence to noncommunicable disease outcomes, the review identifies consistent protective associations, methodological limitations, and key knowledge gaps, and outlines priorities to advance the field and its translation into dietary guidance. It further underscores the need for standardized product characterization and well-powered clinical trials to establish causality. Overall, this work provides the most current and integrative assessment of fermented foods and human health, highlighting their potential as a valuable yet underutilized component of strategies for chronic disease prevention and public health policy.

**Statement of Significance**

This review offers the most comprehensive synthesis to date of observational and interventional research on fermented foods and human health, integrating findings across multiple health domains, identifying critical knowledge gaps, and outlining priorities to advance the field.

## Introduction

Fermented foods have contributed to human nutrition for several millennia. What emerged as spontaneous processes driven by microbial metabolism evolved into intentional practices that persist in cultures across the globe. Food fermentation is known for revolutionizing food storage abilities, transforming flavors, and altering textures, but has recently gained increased public and scientific attention for its potential to impart health-promoting characteristics to foods. However, possible links between consumption of fermented foods, the roles of microorganisms that facilitate their transformations, and human health outcomes remain incompletely understood but are a topic of active investigation. The field of fermented foods research is broad, encompassing a wide variety of food types, health outcomes, and methodologies ranging from observational to interventional studies. Large cohort studies consistently associate the consumption of fermented foods with reduced risk of metabolic disorders, certain cancers, and neurodegenerative diseases. In contrast, most interventional studies are small in scale, often serving as pilot investigations. These studies typically use nonstandardized products, focus on specific health markers, and involve short follow-up periods. Although more rigorous research is needed, the overall body of evidence supports the health-promoting effects of fermented foods with few associated risks. Most concerns relate to the presence of undesirable metabolites, such as alcohol, or unsafe preparation practices. Despite the promising findings, important gaps remain.

This narrative review serves to synthesize and critically evaluate the current evidence on fermented foods and health outcomes. Due to the length of the review, we decided not to include a detailed discussion of studies investigating fermented foods that do not contain viable microbes at the time of consumption due to cooking (e.g., tempeh or sourdough bread) although their potential benefits are briefly described. Clinical evidence was gathered through literature searches conducted in PubMed, Web of Science (Clarivate), and Google Scholar. Where possible, searches were refined to prioritize randomized controlled trials (RCTs), systematic reviews and meta-analyses. Because this is a narrative rather than a systematic review, the search strategy was not exhaustive, and the possibility of selection bias must be acknowledged as a limitation. These insights provide context for future research and funding priorities, helping to build a stronger evidence base that may ultimately inform the development of regulatory frameworks, clinical recommendations, and dietary guidelines.

## Defining Fermented Foods

Fermented foods encompass an impressive array of products as they broadly relate to the activity of transforming edible products by microbial metabolism. Fermented food production involves a variety of starting materials and microbial strains, each driving distinct biochemical transformations. These microbial processes not only contribute to taste and preservation but also generate functional compounds with potential health benefits. Not all fermented products contain viable microbes at the point of consumption or have health-promoting properties. Nevertheless, this does not mean that such foods, or even microbe-free products derived from microbial fermentation, are without potential benefit.

**FIGURE 1:**
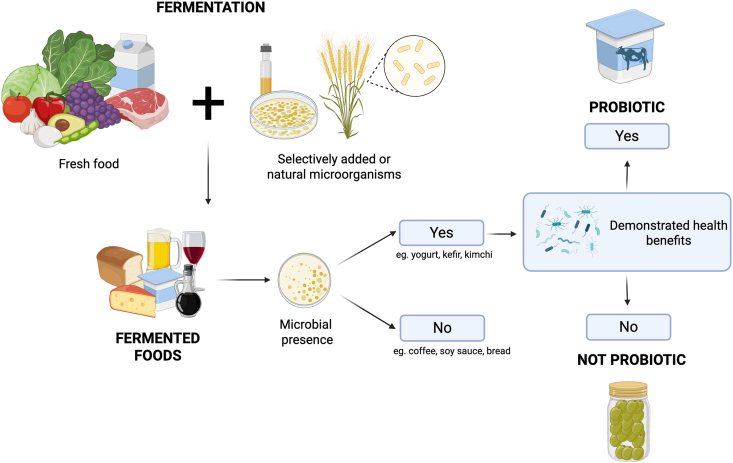
Classification of fermented foods and probiotics based on microbial viability and health benefits. Microorganisms like bacteria, yeast and fungi are naturally present or intentionally added to fresh food products. In microbial fermentation, these organisms convert sugars and other compounds in fresh foods into acids, gases, or alcohol, resulting in fermented food products. Some fermented foods undergo further processing (e.g., pasteurization, filtration) that remove or kill live microorganisms, whereas others contain live microorganisms. Fermented foods that contain live documented probiotic strains in amounts demonstrated to provide a health benefit can be considered probiotic-fermented foods.

With increased interest in the consumption and study of these foods in the modern day, the International Scientific Association for Probiotics and Prebiotics put forward the following consensus definition of fermented foods and beverages: “foods made through desired microbial growth and enzymatic conversions of food components” [1]. Moreover, as described in Figure 1, fermented foods can be categorized as having live microorganisms present (e.g., yogurt, kefir, and traditional kimchi) or having no live microorganisms present (e.g., salami, coffee, and soy sauce) [1]. The microorganisms involved may arise naturally during fermentation (e.g., lactic acid bacteria in sauerkraut) or be deliberately introduced to initiate or guide the fermentative process (e.g., the Symbiotic Culture of Bacteria and Yeast in kombucha). Among the fermented foods that include live microorganisms, only those that contain live documented probiotic strains in amounts demonstrated to provide a health benefit can be considered probiotic fermented foods [1]. Sometimes, probiotic strains are not involved in the fermentation process itself but are instead added to the final product. Recognizing this distinction is particularly important when interpreting findings from clinical studies assessing health outcomes or exploring mechanistic explanations, as misclassification could lead to inaccurate conclusions about the role of specific microbes compared with fermentation processes in driving observed effects.

The global diversity of fermented foods makes it difficult to compile an exhaustive list of those that have been intentionally consumed historically. There are thousands of fermented foods and beverages consumed globally [2]. However, most foods will fall into one of the following categories: leavened cereal products, fruits and vegetables, dairy, soybeans, meats or fish, cereals or grains, nonalcoholic beverages, and alcoholic beverages [2]. Each fermentation may have a unique procedure that helps select for the microbes necessary to complete the food’s transformation through variables such as time, temperature, salinity, or moisture. To systematically categorize these diverse products, Gänzle et al. [3] mapped fermented foods onto a “periodic table” of fermentation, highlighting the wide range of microbial and substrate combinations used across global food traditions.

In the context of food, fermentation extends beyond the traditional biochemical definition to include both anaerobic and aerobic metabolism performed by bacteria, fungi, and yeast [1]. Although in some scientific contexts, fermentation is defined strictly as an anaerobic process, in food science, the term is used more broadly to capture the diverse microbial transformations that contribute to flavor, texture, and preservation. The most common microbes involved in food fermentations include lactic acid bacteria (LAB), acetic acid bacteria (AAB), bacilli, yeasts, and filamentous fungi [1]. LAB are key drivers of the transformation necessary for a multitude of common fermented foods, including fermented milk products, vegetables, sausages, and sourdoughs [4]. AAB are known best for their ability to withstand low pH while metabolizing ethanol into acetic acid, driving vinegar production and playing a role in the fermentative process of kombucha where they convert yeast-generated ethanol and glucose into acetic and glycolic acids, respectively, contributing to the unique composition and flavor of the beverage [5,6]. Through the production of bioactive peptides and hydrolysates via specialized enzymatic activity, *Bacillus* spp. play an important role in many alkaline legume-based fermentations, and have been detected in seafood fermentations as well [7,8]. Filamentous fungi such as *Aspergillus oryzae* or *Rhizopus oligosporus* are commonly used in soy fermentations, producing tempeh, Quorn meat substitutes, and koji (a key intermediate product to foods like soy sauce or miso) [9,10]. The presence of these microbes under specific conditions allows for the fermentative transformation of food in ways that have supported human nourishment throughout history while fending off food spoilage, poisoning or intoxication.

## Clinical Evidence Supporting the Health Effects of Fermented Foods

**FIGURE 2:**
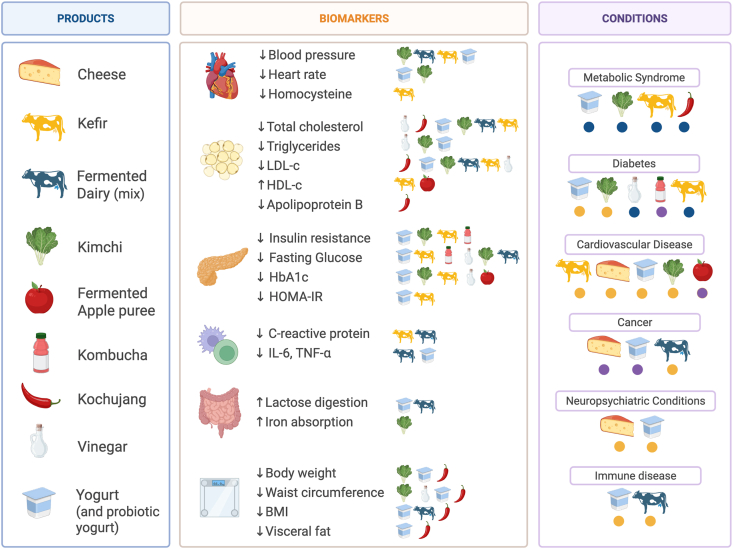
The impact of various fermented foods on biomarkers of metabolic health and the associated decreased disease risks. The left column lists the food products to which effects on biomarkers or disease risks have been reported in the literature. The middle column indicates reported changes in biomarkers, with symbols indicating which fermented foods were associated with improved biomarkers. The right column shows reported decreases in disease risks associated with specific fermented food products. The circles indicate the level of evidence based on all studies summarized in Table 1. Blue represents stronger evidence (including large RCTs, systematic reviews, and meta-analyses); **yellow** indicates moderate evidence (small RCTs or inconsistent findings); and **purple** denotes the lowest level of evidence (primarily from cohort and case-control studies). HbA1c, hemoglobin A1c; RCT, randomized controlled trial.

Despite the presence of fermented foods in human dietary patterns for millennia, research techniques have only recently enabled scientists to explore the microbes and mechanisms behind fermented food preservation and their support of human nutrition and health. Drawing on the ubiquitous nature of fermented foods and our developing understanding of the community of bacteria, fungi, and viruses that reside in the human gastrointestinal tract, known as the gut microbiota [11], both public and scientific audiences have developed a greater interest in understanding the ways that these foods may influence health [1]. Changes to lifestyle, dietary patterns, and increased antibiotic exposure compared with preindustrial times contribute to alterations in the human gut microbiome, often leading to decreased microbial diversity, loss of key taxa, reduced functional capacity, and inflammation [12,13]. These microbial shifts are also hypothesized to contribute to the greater prevalence of noncommunicable diseases (NCDs) in modern societies, including many immunologic, metabolic, and neurologic diseases [12,13]. Diseases with associated gut microbiome alterations include osteoarthritis [14], diabetes [15], obesity [16,17], depression [18], Parkinson’s disease [19], and cancers [20], among others. Improving the gut microbiome and reducing inflammation through dietary inputs like fermented foods may therefore be a path to improving human health status (Figure 2). This potential has gained global traction in fermented food research in recent decades, so the existing evidence for the role of fermented foods in health and disease prevention will now be reviewed, and the many areas for continued or improved research will be introduced. See Table 1 [[21], [22], [23], [24], [25], [26], [27], [28], [29], [30], [31], [32], [33], [34], [35], [36], [37], [38], [39], [40], [41], [42], [43], [44], [45], [46], [47], [48], [49], [50], [51], [52], [53], [54], [55], [56], [57], [58], [59], [60], [61], [62], [63], [64], [65], [66], [67], [68], [69], [70], [71], [72], [73], [74], [75], [76], [77], [78]] for a summary of the studies discussed.

**TABLE 1:** Summary of reviewed studies

Fermented dairy products and metabolic health					
Food	Study type	Health condition	Summary findings	Biomarker	Reference
Yogurt	Cross-sectional observational	Diet quality and metabolic profiles in children	Yogurt consumption linked with better diet quality and improved metabolic profiles in children	↓Fasting insulin; ↓HOMA-IR; ↑QUICKI	Zhu et al., 2015 [21]
Yogurt	Cross-sectional observational	Human gut microbiome	Yogurt consumption associated with reduced visceral fat mass, shifts in gut microbiota and metabolome	↓Visceral fat mass	Le Roy et al., 2022 [22]
Yogurt (dietary exposure)	Prospective cohort study	Metabolic syndrome	Higher yogurt intake associated with reduced risk of metabolic syndrome	↓Waist circumference: (lower abdominal obesity in higher yogurt consumers) ↓Fasting plasma glucose ↓Serum triglycerides ↑HDL cholesterol ↓Blood pressure (SBP/DBP)	Cheraghi et al., 2016 [23]
Dairy (mixed, not limited to yogurt)	Meta-analysis of cohort studies	Type 2 diabetes	Dose–response: higher dairy intake associated with lower T2DM risk	Not assessed	Gijsbers et al., 2016 [24]
Dairy (milk, yogurt, etc.)	Systematic review and dose–response meta-analysis of cohort studies	Type 2 diabetes	Higher dairy intake associated with lower T2DM risk	Not assessed	Aune et al., 2013 [25]
Fermented dairy foods	Meta-analysis of observational studies	Type 2 diabetes	Dose-dependent inverse association between fermented dairy intake and T2DM risk	Not assessed	Zhang et al., 2022 [26]
Yogurt/cultured fermented milk	Systematic review (mixed RCTs and cohorts)	Various health outcomes	On the basis of reviewed studies, a causal relationship exists between lactose digestion and tolerance and yogurt consumption. Generally favorable associations for fermented dairy on several health outcomes	Not assessed	Savaiano et al., 2021 [27]
Dairy products (general)	Systematic review of RCTs	Inflammation	Evidence suggests dairy products (esp. fermented) has anti-inflammatory effects, but heterogeneous	Not assessed	Bordoni et al., 2017 [28]
Probiotic yogurt	Randomized controlled trial	Type 2 diabetes mellitus	Improved glycemic control and lipid profile in patients with T2DM	After adjustment, probiotic group showed ↓ HbA1c, ↓TC, ↓ LDL-c; no significant change in TG or HDL-c; trend toward ↓ FPG (nonsignificant)	Mirjalil et al., 2023 [29]
Functional yogurt (NY-YP901)	Randomized controlled trial	Metabolic syndrome	Improved metabolic syndrome parameters vs. control	↓Body weight, ↓ BMI, ↓ LDL-c; no significant change in fasting glucose, HbA1c, BP, TC, HDL-c, TG	Chang et al., 2011 [30]
Fermented milk	Double-blind RCT	Type 2 diabetes mellitus	Ingestion of milk fermented with *L. helveticus* compared with placebo for 12 wk did not significantly reduce BP in patients with T2DM	No significant difference vs. placebo in BP, HbA1c, lipids, CRP, PAI-1, TNF-α, tPA-Ag, vWF-Ag; ↓Daytime and 24-h heart rate (HR); fasting glucose change favored fermented milk over placebo (smaller increase in treatment)	Hove et al., 2015 [31]
Probiotic vs. conventional yogurt	Randomized controlled trial	Weight loss in females with obesity on energy-restricted diet	Probiotic yogurt led to greater weight and fat mass reduction	↓TC, ↓ LDL-C, ↓ HOMA-IR, ↓ 2-h glucose, ↓ fasting insulin; no significant change in FPG, HDL-c, TG	Madjd et al., 2016 [32]
Probiotic yogurt	Randomized controlled trial	Nonalcoholic fatty liver disease (NAFLD)	Probiotic yogurt consumption improved BMI and insulin levels	↓Weight and BMI; ↓Fasting insulin; no significant change in serum leptin or adiponectin; HOMA-IR also did not change significantly	Nabavi et al., 2015 [33]
Yogurt/dairy	Umbrella review and updated meta-analyses of cohorts	Cardiovascular disease/CVD risk	Yogurt consumption associated with modest inverse association with CVD (RR = 0.92)	↓CVD risk, ↑ BMD; fracture risk mixed; limited effect on bone resorption markers	Sharifan et al., 2025 [34]
Kefir	Randomized controlled trial	Metabolic syndrome	Kefir improved blood parameters and reduced cardiovascular risk markers	↓Triglycerides and LDL cholesterol, ↑ HDL cholesterol ↓Oxidized LDL cholesterol ↓hs-CRP (inflammation) No change: liver/kidney markers, fasting glucose, HbA1c ↓Cardiovascular risk (Framingham score)	Ghizi et al., 2021 [35]
Probiotic fermented milk (*Bifidobacterium lactis*)	Randomized controlled trial	Metabolic syndrome	Improved lipid profile and cytokines in patients with metabolic syndrome	↓TC and LDL-c ↓TNF-α and IL-6 (inflammatory cytokines) ↓BMI	Bernini et al., 2016 [36]
Kefir	Systematic review of RCTs	Various conditions (e.g., metabolic, GI, oral health)	Review concluded kefir may benefit metabolic and gut health but evidence quality generally low–moderate	Reduced oral Streptococcus mutans levels	Kairey et al., 2023 [37]
Probiotic fermented milk (Kefir)	Randomized double-blind placebo-controlled trial	Type 2 diabetes	Improved glycemic control and lipid profile compared with placebo	↓HbA1c ↓Fasting blood glucose: notable decrease in the kefir group. No significant change: serum triglycerides, TC, LDL-c, and HDL-c levels remained unaffected in both groups.	Ostadrahimi et al., 2015 [38]
Probiotic fermented milk (Kefir)	Randomized controlled trial	Type 2 diabetes	Reduced serum insulin and homocysteine levels in patients with T2DM	↓HOMA-IR (improved insulin sensitivity) ↓Homocysteine No change: serum insulin	Alihosseini et al., 2017 [39]
Kefir	Parallel-group randomized controlled study	Metabolic syndrome	Regular kefir consumption altered gut microbiota composition in patients with metabolic syndrome	Significant reductions in fasting insulin, HOMA-IR, TNF-α, IFN-γ, and both systolic and diastolic blood pressure	Bellikci-Koyu et al., 2019 [40]
Kefir	Systematic review and meta-analysis of RCTs	Glycemic control in individuals with/at risk for type 2 diabetes	Kefir consumption significantly improved fasting blood glucose and HbA1c compared with control	↓Fasting blood sugar ↓Insulin levels No change: HbA1c	Salari et al., 2021 [41]
Botanical fermented foods and metabolic health					
Kombucha	Randomized, placebo-controlled, crossover trial	Glycemic control (postmeal response)	Consumption of live kombucha with a standard carb meal lowered postmeal glycemic and insulin response compared with placebo	↓Glycemic index (68 vs. 84–86) ↓Insulin index (70 vs. 81–85)	Atkinson et al., 2023 [42]
Kombucha	Pilot randomized, placebo-controlled study	Glycemic control in T2DM	Kombucha was associated with reduced fasting glucose in people with T2DM	↓Fasting blood glucose from 164 to 116 mg/dL	Mendelson et al., 2023 [43]
Kombucha	Systematic review of empirical human evidence	Broad human health outcomes	Evidence from human studies remains limited and inconsistent; most claims not yet strongly supported	Not assessed	Kapp and Sumner, 2019 [44]
Kochujang (fermented soybean-based red pepper paste)	Randomized controlled trial	Overweight adults (visceral fat and lipid profile)	Decreased visceral fat and improved blood lipid profiles	↓Visceral fat Body weight and WHR: no change ↓Triglycerides and ApoB vs. placebo	Cha et al., 2013 [45]
*Aspergillus oryzae*-fermented Kochujang	Randomized controlled trial	Hyperlipidemia	Aspergillus oryzae-fermented kochujang lowered serum cholesterol levels	↓TC (215.5 → 194.5 mg/dL) ↓LDL-c: trend, not significant No change: HDL-c, triglycerides	Lim et al., 2015 [46]
Traditional and commercial Kochujang	Randomized controlled trial	Adults with overweight/obesity	Both traditional and commercial kochujang reduced body weight and improved obesity-related markers	↓Waist circumference: HTK and CK ↓Visceral fat: HTK Improved lipid profile: HTK and LTK ↑Beneficial gut microbes: all groups	Han et al., 2022 [47]
Fresh and fermented Kimchi	Randomized controlled trial	Prediabetic individuals	Beneficial effects on glycemic control and metabolic health vs. fresh kimchi	↓Body weight, BMI, waist circumference: fresh and fermented ↑Insulin sensitivity and ↓ insulin resistance ↓Blood pressure Improved glucose tolerance: 33% fermented vs. 9.5% fresh	An et al., 2013 [48]
Fermented Kimchi	Systematic review and meta-analysis of intervention and prospective cohort studies	Cardiometabolic indicators	Regular consumption associated with improved anthropometric and cardiometabolic markers Prospective studies: lower risk of metabolic syndrome, cancer, and higher chance of normal body weight	↓Fasting glucose ↓Triglycerides ↓Blood pressure	Ahn et al., 2025 [49]
Korean traditional diet (includes fermented foods)	Randomized controlled trial	Hypertensive and type 2 diabetic patients	Improved blood pressure and metabolic outcomes	↓HbA1c ↓Heart rate: −7.1 bpm ↓BMI, body fat, waist-to-hip ratio	Jung et al., 2014 [50]
Vinegar consumption	Systematic review and meta-analysis of intervention studies	Type 2 diabetes	Vinegar intake improved glycemic control in adults with T2DM	↓Fasting glucose ↓HbA1c ↓TC and LDL-c	Cheng et al., 2020 [51]
Apple cider vinegar	Randomized placebo-controlled trial	Type 2 diabetes with poor glycemic control	Improved glycemic control compared with placebo	↓HbA1c and fasting glucose ↓TC and triglycerides ↓Waist-to-hip ratio No change: LDL-c, HDL-c, body weight	Kausar et al., 2019 [52]
Dairy fermented foods and cardiovascular health					
Fermented dairy foods	Meta-analysis of cohort studies	Cardiovascular diseases	Higher intake of fermented dairy associated with lower CVD risk Cheese: OR = 0.87, Yogurt: OR = 0.78	Not assessed	Zhang et al., 2020 [53]
Cheese	Meta-analysis of observational studies	All-cause mortality, major cardiovascular outcomes, several chronic diseases (including type 2 diabetes and dementia), and certain cancers	Cheese consumption was linked to modestly lower risks of all-cause mortality, major cardiovascular outcomes, several chronic diseases (including type 2 diabetes and dementia), and certain cancers.	Not assessed	Luo et al., 2023 [54]
Milk and fermented milk	Longitudinal cohort study	Stroke risk	Fermented milk consumption inversely associated with stroke risk	Not assessed	Olsson et al., 2022 [55]
Probiotic fermented milk products	Systematic review and meta-analysis of RCTs	Blood lipid concentrations	Probiotic fermented milk products improved blood lipid profiles	↓TC and LDL-c No change: HDL-c, triglycerides Better effects: males, ≥8 wk consumption	Ziaei et al., 2021 [56]
Dairy intake (including fermented)	Systematic review with network meta-analysis	Cardiometabolic health markers	Certain fermented dairy types linked to improved cardiometabolic markers; network meta-analysis supports differential effects by dairy type	↓Systolic BP: 5–8 mmHg ↑Fasting glucose and HbA1c No change: body weight, BMI, waist, lipids	Kiesswetter et al., 2023 [57]
*Lactobacillus helveticus-*fermented milk	Randomized controlled trial	Hypertension	Lowered 24-h ambulatory blood pressure in hypertensive subjects	↓Systolic BP, ↓ Diastolic BP (24-h ambulatory) No change: body weight, lipids, glucose, safety labs	Jauhiainen et al., 2005 [58]
Fermented milk product	Randomized controlled trial	Hypercholesterolemia in healthy middle-aged males	Hypocholesterolemic effect demonstrated	↓Total cholesterol, ↓LDL-c No change: HDL-c, triglycerides, body weight, safety labs	Agerbaek et al., 1995 [59]
Fermented milk product	Randomized placebo-controlled, double-blind trial	Plasma lipoproteins in adults	Six-month consumption lowered plasma lipoprotein levels	↓TC, ↓LDL-c No change: HDL-c, triglycerides, body weight	Richelsen et al., 1996 [60]
Global dairy consumption (fermented and nonfermented)	Global pooled cohort analysis	Incident cardiovascular disease	Higher dairy consumption associated with lower risk of CVD in global pooled analysis	Not assessed	Zhuang et al., 2025 [61]
Dairy (overall, including fermented)	Scoping review of systematic reviews and meta-analyses	Multiple health outcomes	Synthesized evidence shows dairy (esp. fermented) often associated with beneficial outcomes. ↓ Risk: cardiovascular disease, some cancers (bladder, breast, colorectal, oral), type 2 diabetes, overweight/obesity, joint health ↑ Risk (few): prostate, ovarian, liver cancers (in limited reports)	Not assessed	Akyil et al., 2025 [62]
Kefir	A systematic review and meta-analysis of randomized controlled trials	Metabolic syndrome	Kefir had a beneficial effect in decreasing insulin resistance; however, no effect was seen on BW, FBS, HbA1C, and lipid profile.	↓Fasting insulin, ↓ HOMA-IR (insulin resistance) No change: TC, LDL cholesterol, HDL cholesterol, triglycerides, fasting blood sugar, HbA1c, body weight	Yahyapoor et al., 2023 [63]
Yogurt	Systematic review and dose–response meta-analysis (cohort studies)	All-cause, CVD, and cancer mortality	High yogurt intake was associated with lower all-cause and cardiovascular mortality, with no significant effect on cancer mortality, and benefits plateaued above ∼0.5 serving/d.	Not assessed	Tutunchi et al., 2023 [64]
Nondairy fermented foods and cardiovascular health					
Tempeh-based beverage	Randomized controlled trial	Hyperlipidemia	Consumption of tempeh beverage reduced LDL cholesterol levels	↓TC, ↓LDL cholesterol, ↓Triglycerides No change: HDL cholesterol	Wirawanti et al., 2017 [65]
Kimchi (fermented vegetable)	Randomized controlled trial	Healthy young adults—serum lipids	Regular kimchi consumption improved serum lipid profiles	↓Total cholesterol, ↓LDL cholesterol, ↓Triglycerides, ↓FBG No change: HDL cholesterol	Choi et al., 2013 [66]
Lactofermented Annurca apple purée	Randomized controlled trial	Plasma lipid levels and oxidative amines	Intake of lactofermented apple purée improved plasma lipid profile and reduced oxidative amine levels	↓HDL cholesterol ↓TMAO No change: total cholesterol, LDL, triglycerides, blood glucose	Tenore et al., 2019 [67]
Association between fermented foods and cancer					
Gut-microbiota-targeted diet (fermented foods vs. fiber diet)	Randomized controlled trial	Immune function in adults	Diets enriched in fermented foods improved microbiota diversity and modulated immune markers	High-fermented-food diet ↑microbiome diversity, ↓19 inflammatory markers (e.g., IL-6).	Wastyk et al., 2021 [68]
Fermented dairy foods (yogurt, cheese)	Meta-analysis (observational studies)	Cancer risk	Higher intake of fermented dairy foods (including yogurt) was associated with lower overall cancer risk (OR ≈ 0.86, cohort studies) and yogurt specifically with reduced risk (OR ≈ 0.87 overall; OR ≈ 0.81 in cohort studies)	Not assessed	Zhang et al., 2019 [69]
Fermented milk	Systematic review	Breast and colorectal cancer	Fermented milk consumption reduced risk of breast and colorectal cancer	Not assessed	Savaiano et al., 2021 [27]
Fermented milk products	Case–control study	Breast cancer	Inverse association between fermented milk consumption and breast cancer risk	Not assessed	van’t Veer et al., 1989 [70]
Impact of fermented foods on mental and cognitive health					
Fermented dairy foods	Meta-analysis of cohort studies	Depressive symptoms	Higher fermented dairy consumption associated with reduced depressive symptoms. Cheese: reduced depression risk (OR = 0.91). Yogurt: decreased depression risk (OR = 0.84).	Not assessed	Luo et al., 2023 [71]
Yogurt (strain OLL1073R-1)	Secondary analysis of RCT	Psychological quality of life in healthcare workers	Consumption of OLL1073R-1 yogurt improved psychological quality of life, ↑ sleep quality (PSQI), ↑ general health and vitality (SF-8) and ↓ constipation (GSRS).	Not assessed	Kinoshita et al., 2021 [72]
Fermented foods and social anxiety	Observational (cross-sectional, interaction model)	Social anxiety and neuroticism	Higher consumption of FFs associated with lower social anxiety in individuals with higher neuroticism	Not assessed	Hilimire et al., 2015 [73]
Fermented foods and prebiotics	Prospective cohort study	Cognitive performance, depressive and anxiety symptoms under stress	FF and prebiotic consumption associated with better cognitive function and fewer depressive/anxiety symptoms in stressed medical students	Not assessed	Karbownik et al., 2022 [74]
Effects of fermented foods on immune function					
High-fiber vs. fermented-food diet	Citizen-science randomized controlled trial	Gut microbiota, immune function, sleep quality	Fermented-food diet improved microbiota diversity and modulated immune responses, distinct from high-fiber diet	High-fermented-food diet ↑ immune markers (CD5, CD6, CD8A, IL-18R1), SIRT2, modest ↑ microbiome diversity in participants >50 resembling younger participants	Belt et al., 2025 [75]
Traditional fermented foods	Observational case–control study	Infantile atopic dermatitis	Consumption of traditional fermented foods associated with lower risk of infantile atopic dermatitis	Not assessed	Celik et al., 2019 [76]
Probiotic yogurt	Systematic review	Maternal health and pregnancy outcomes	Probiotic yogurt associated with benefits for maternal metabolic and obstetric outcomes (metabolic and inflammatory)	↓Oxidative stress markers, ↓ inflammatory markers, ↑ serum calcium, and improved metabolic biomarkers related to gestational diabetes risk.	He et al., 2020 [77]
Probiotic fermented dairy products	Systematic review and meta-analysis of RCTs	Incidence of respiratory tract infections	Probiotic fermented dairy consumption reduced incidence of respiratory tract infections. Stronger effects for Lactobacillus-containing products.	Not assessed	Rashidi et al., 2021 [78]

Abbreviations: ApoB, apolipoprotein B; BP, blood pressure; bpm, beats/min; CI, confidence interval; DBP, diastolic blood pressure; FBG, fasting blood glucose; FF, fermented food; FPG, fasting plasma glucose; GI, gastrointestinal; GSRS, Gastrointestinal Symptom Rating Scale; HbA1c, hemoglobin A1c; HR, heart rate; hs-CRP, high-sensitivity C-reactive protein; IFN-γ, interferon-gamma; OR, odds ratio; PAI-1, plasminogen activator inhibitor-1; PSQI, Pittsburgh Sleep Quality Index; QUICKI, Quantitative Insulin Sensitivity Check Index; RCT, randomized controlled trial; RR, relative risk; SBP, systolic blood pressure; SIRT2, Sirtuin 2; SF-8, Short Form-8 Health Survey; TC, total cholesterol; TG, triglycerides; TMAO, trimethylamine N-oxide; Tregs, regulatory T cells; tPA-Ag, tissue plasminogen activator antigen; vWF-Ag, von Willebrand Factor Antigen; WHR, waist-to-hip ratio.

### Impact of fermented foods on metabolic and cardiovascular health

Metabolic health refers to the optimal functioning of processes that regulate blood glucose, lipid concentrations, blood pressure, and body composition, and its disruption is closely linked to increased risk of chronic diseases. Type 2 diabetes mellitus (T2DM) is a metabolic condition influenced by both genetic and environmental risk factors that impair blood glucose regulation [79]. A major contributor to T2DM risk is the modern “western diet,” typically low in fiber and high in saturated fat and processed meat [80,81]. Consequently, researchers have increasingly focused on dietary strategies to prevent and manage the disease [82]. Metabolic syndrome (MetS), which is a cluster of comorbid conditions including central adiposity, hypertension, and disordered glucose and lipid metabolism, shares many characteristics with T2DM and often co-occurs with it [83]. MetS itself is strongly associated with cardiometabolic diseases [cardiovascular disease (CVD)] too. Given its link to highly prevalent NCDs, reducing the risk and severity of T2DM and MetS is an important public health objective, and novel dietary strategies may play a critical role. The current evidence indicates that fermented foods may improve glycemic control, reduce fasting blood glucose, and influence additional aspects of metabolic health. These conclusions are drawn from both observational and interventional studies, which collectively highlight the potential of fermented foods to reduce the risk of T2DM and to improve metabolic parameters relevant to MetS and CVD. The following sections provide a more detailed overview of this body of research, from observational to interventional studies (Table 1). Additionally, Figure 2 summarizes the associations between the reviewed fermented foods and health outcomes and related health biomarkers.

#### Fermented dairy products and metabolic health

Compared with other fermented food groups, fermented dairy products have been studied more extensively in relation to MetS, T2DM, and related NCDs. A recent 2024 meta-analysis of prospective cohort studies reported a nonlinear inverse association between total dairy intake and prediabetes risk, with the lowest risk at ∼3.4 servings/d [relative risk (RR): 0.75, 95% confidence interval (CI): 0.60, 0.93]. Total and high-fat cheese intake also showed nonlinear protective associations, lowest at ∼2.1 servings/d, although risk increased at intakes >4 servings/d [84]. Overall, moderate dairy-fermented food consumption may lower prediabetes risk, but heterogeneity in study design, potential reverse causation, and residual confounding warrant cautious interpretation and further research. The following section summarizes the evidence from both observational and interventional studies focusing specifically on yogurt and kefir intake.

##### Yogurt and metabolic health

Yogurt is the most common fermented dairy product consumed globally and the most well studied. Yogurt intake has been linked to better overall diet quality in both adults and children [21,85], and to reduced visceral fat and healthier dietary scores [22]. Moreover, a prospective cohort study of 3616 healthy adults reported that yogurt consumption significantly reduced the risk of developing MetS over 2 y [odds ratio (OR): 0.43; 95% CI: 0.18, 1.01] [23]. The authors estimated that each daily serving of yogurt (200 g, 1 single-serve container) was associated with a 57% decrease in incidence [23]. Recently, a United Kingdom twin study showed that yogurt consumption also coincided with transient increases in *Streptococcus thermophilus* and *Bifidobacterium animalis subsp. lactis* in feces, microbes commonly found in yogurt cultures, suggesting a role for the gut microbiome in mediating effects [55]. In a 2015 meta-analysis of observational studies investigating the relationship between T2DM and dairy intake, researchers found a strong inverse relationship between yogurt intake and T2DM through analysis of 11 included studies [24]. The risk of T2DM was found to be decreased by 14% when yogurt intake was between 80 and 125 g/d compared with 0 g/d [24]. These data corroborate findings from a 2013 meta-analysis showing an inverse relationship between yogurt intake and T2DM risk, which plateaus at ∼120 to 140 g/d [25]. A 2022 meta-analysis of 15 cohort and case-control studies involving 485,992 participants found that higher intake of fermented dairy foods was associated with reduced diabetes risk (OR: 0.925; 95% CI: 0.856, 0.999), with yogurt showing an even stronger protective effect (OR: 0.828; 95% CI: 0.729, 0.941) [26].

A systematic review by Savaiano et al. [27] and a meta-analysis by Zhang et al. [26], continue to support yogurt’s dose-dependent protective effect against T2DM. Analysis of 5124 children aged 2–18 in the NHANES study also indicated that more frequent consumption of yogurt was associated with lower HOMA-IR values, lower fasting insulin concentrations, and better Quantitative Insulin Sensitivity Check Index (QUICKI) scores [21]. Savaiano et al. [27] concluded that consumption of fermented dairy is consistently associated with a lower risk of T2DM and improved disease-related outcomes. It is important to note that comparators varied across studies, including ultraheated yogurt, nonfermented dairy products, milk, chemically fermented dairy, pasteurized yogurt, and no yogurt as these differences can influence study outcomes and complicate direct comparisons. Another systematic review that assessed inflammation-modulating properties in 52 studies reported that fermented dairy had significant anti-inflammatory effects, both in general and compared with nonfermented dairy [28]. Notably, the anti-inflammatory effects were especially pronounced in individuals with MetS, likely because this population often exhibits elevated baseline concentrations of systemic inflammation, including higher circulating C-reactive protein (CRP), IL-6, and TNF [28]. Reductions in these markers may be associated with improved pathophysiological features of MetS.

A growing body of trials has also investigated probiotic-enriched yogurts. In 1 RCT, 60 participants consumed either 200 g/d of probiotic yogurt containing 4.65 × 10^6^ CFU/g *Lactobacillus acidophilus* and *B. animalis subsp. lactis* or conventional yogurt. The study found that after the 12-wk intervention, when baseline covariates were adjusted for, the probiotic yogurt group experienced a greater reduction in hemoglobin A1c (HbA1c), total cholesterol, and low-density lipoprotein (LDL) cholesterol ("bad" cholesterol) compared with the conventional yogurt group [29]. HbA1c reflects the mean blood glucose concentrations over the past 2–3 mo, and is commonly used to diagnose and monitor diabetes. Notably, mean change in HbA1c and fasting glucose concentrations showed a trend to decrease in both yogurt-consuming groups postintervention [29]. An 8-wk study with 101 participants found that functional yogurt (containing *S. thermophilus*, *L. acidophilus*, *B. infantis, B. breve* CBG-C2, and *Enterococcus faecalis* FK-23) consumption decreased LDL cholesterol and body weight compared with placebo [30]. In individuals with T2DM, a 12-wk trial using yogurt fermented with *L. helveticus* demonstrated improvements in heart rate and fasting plasma glucose relative to acidified milk controls [31]. Other RCTs have reported that probiotic yogurts lowered LDL cholesterol, total cholesterol, HOMA-IR, and fasting insulin concentrations more effectively than conventional yogurts in individuals with obesity or nonalcoholic fatty liver disease [now called metabolic dysfunction-associated steatotic liver disease] [32,33]. Collectively, these findings indicate that probiotic strains may enhance the health-promoting effects of fermented dairy, although traditional yogurts still confer benefits.

Evidence strongly supports yogurt as beneficial for metabolic health. Large-scale meta-analyses and cohort studies show that higher yogurt intake is associated with reduced risk of type 2 diabetes, MetS, and visceral adiposity, with benefits observed even at moderate intakes. Yogurt consumption is also linked to improved insulin sensitivity, healthier dietary patterns, and favorable shifts in gut microbiota, suggesting both behavioral and biological mechanisms. Probiotic-enriched yogurts may offer additional advantages, although conventional yogurts also confer significant benefits. Although most data are observational, the consistent, dose-dependent associations provide compelling support for yogurt as a protective dietary component. Future RCTs are needed to confirm causality and clarify the roles of specific probiotic strains, fermentation processes, and comparator foods.

##### Kefir and metabolic health

Other fermented milk products such as kefir also show potential to improve metabolic health. A 2021 meta-analysis of 6 RCTs with kefir found that intervention groups experienced a greater reduction in fasting blood glucose and insulin than control groups. However, HbA1c concentrations were not significantly different between groups [41]. Although the trials were small, the limited improvements in glycemic control indicate a potential role for Kefir consumption in metabolic health. In a 12-wk double-blind RCT of 48 individuals with obesity, kefir consumption reduced blood pressure, fasting glucose, LDL cholesterol, non-HDL cholesterol, triglycerides (TGs), and oxidized LDL, while raising high-density lipoprotein (HDL) cholesterol ("good" cholesterol) in females [35]. Notably, non-HDL cholesterol encompasses LDL, very-low density lipoprotein (VLDL), intermediate-density lipoprotein (IDL), and lipoprotein, and is regarded as a stronger predictor of cardiovascular disease risk than LDL cholesterol alone. These results therefore provide compelling evidence for the cardioprotective potential of kefir consumption. Additionally, participants in the kefir group exhibited a lower Framingham Risk Score, indicating a reduced 10-y risk of cardiovascular events. Interestingly, increases in HDL cholesterol concentrations were observed only in females. Another study supporting these findings is an RCT involving 51 individuals with obesity reported that milk fermented with *B*. *animalis subsp. lactis* HN019 reduced total cholesterol, LDL cholesterol, TNF, IL-6, and BMI [36].

A double-blind RCT involving 60 individuals with T2DM compared the effects of consuming either 600 mL/d of fermented milk enriched with probiotic strains (*L. casei*, *L. acidophilus*, and *B. animalis* subsp. *lactis*), which the authors referred to as kefir, or 600 mL/d of conventional fermented milk containing *S. thermophilus* and *L. bulgaricus*, referred to as dough. Both interventions were found to improve multiple diabetes-related biomarkers [38]. The kefir group showed a significant reduction in HbA1c compared with the dough group, and fasting blood glucose concentrations were better in the kefir group compared with the dough after 8 wk [38]. In additional analyses, the researchers reported that HOMA-IR values were improved after the kefir intervention and compared with the dough group [39]. However, inclusion of a proper control group receiving nonfermented milk would be important to account for potential placebo effects [39]. In another study, individuals with MetS, 12 wk of 180 mL daily kefir consumption resulted in a significant decrease in HOMA-IR values. However, this reduction was not significantly different from the control group, who consumed 180 mL of milk per day, which may partly reflect the limited sample size (*n =* 12 intervention, *n =* 10 control) [40].

Although these findings are promising, a 2023 systematic review by Kairey et al. [37], which summarized findings of 16 existing nonprobiotic-supplemented kefir RCTs, pointed out that the body of evidence is characterized by studies with low sample sizes and high risk of bias. Differences in kefir preparation methods also contribute to variability in health outcomes. Some studies use kefir prepared with traditional kefir grains, whereas others use commercially available kefir, which may lack characteristic kefir organisms. A recent pilot randomized crossover trial in males with mildly elevated cholesterol showed that traditional kefir reduced LDL cholesterol and markers of inflammation and endothelial function, whereas commercial kefir did not [32]. These findings underscore the importance of product composition and microbial authenticity in determining fermented food benefits, highlighting a key limitation when replicating results or conducting systematic reviews, as products labeled identically (e.g., “kefir”) can have distinct microbial profiles and health effects.

Overall, current clinical evidence on kefir consumption indicates a supportive role in glycemic control and metabolic health. Both meta-analyses and individual interventional studies report improvements in markers such as fasting blood glucose, insulin, HOMA-IR, and blood lipid concentrations. Despite this evidence, larger interventional studies, well-reported kefir microbial characteristics, and efforts to isolate kefir intake in diverse prospective cohorts would further clarify kefir’s protective potential and fortify current evidence.

#### Botanical fermented foods and metabolic health

Another class of fermented foods currently being explored for potential to attenuate MetS risk is botanical fermented foods (BFF) [86,87]. This class includes fermented fruits, vegetables, nuts, cereals, and pulses. Common fermented foods such as tempeh, kimchi, and sauerkraut would be categorized as BFFs. Findings from existing RCTs on BFFs were recently discussed in a systematic review conducted by Chan et al. [87]. This systematic review highlighted the limited RCTs available on BFFs, while concluding that most of the included fermented soy and vegetable products were associated with an improvement in glycemic, anthropometric, and/or inflammatory parameters [87]. Considering that elevated fasting blood glucose is a criterion that can be shared between T2DM and MetS, many of the potential benefits that are observed for blood glucose control with the consumption of foods such as kimchi, kombucha, and vinegar extend to the context of MetS as well and are reviewed in the following sections.

##### Kombucha and metabolic health

Despite the presence of kombucha on store shelves and popular belief in its health-promoting qualities, the scientific evidence to support its consumption rests heavily on in vitro data and animal experimental models [44]. A systematic review by Kapp et al. [44], which predated the recent Atkinson et al. [42] trial, reported that no controlled human trials investigating kombucha had been published up to July 2018. To our knowledge only 1 study, a randomized placebo-controlled crossover trial by Atkinson et al. [42] recently investigated differences in postmeal (2 h) glycemic index response and insulin index when 11 adults with normal glucose tolerance and BMI consumed a standardized high glycemic index meal of rice and peas with either kombucha, lemonade, or soda water. Results demonstrated lower glycemic index and insulin index responses when the meal was eaten alongside kombucha compared with either lemonade or soda water, with no significance found between the non-kombucha conditions [42]. Although promising, these findings are limited to metabolically healthy participants, and further studies are needed in populations with T2DM or MetS to determine reproducibility and clinical relevance. Progress is underway, as a randomized controlled pilot published in 2023 investigated the antihyperglycemic activities of kombucha in adults with T2DM. The study showed that kombucha lowered mean fasting blood glucose concentrations at 4 wk compared with baseline (164 compared with 116 mg/dL, *P <* 0.035), whereas the placebo did not (162 compared with 141 mg/dL, *P <* 0.078) [43]. These findings provide preliminary evidence that requires confirmation in larger studies but suggest a promising role for BFFs, such as kombucha, in modulating postprandial glycemic responses and potentially contributing to the management of T2DM. Overall, the evidence for kombucha’s metabolic effects is very limited, based primarily on preclinical studies with a few small human RCT, highlighting the need for larger trials and in relevant T2DM/MetS populations rather than healthy participant.

##### Kochujang and metabolic health

A fermented soy and pepper-based product popularized in Korea called kochujang has similarly been shown to result in improvements in lipid profiles in the limited number of existing RCTs. Compared with placebo, 60 males and females with overweight who consumed 32 g/d kochujang in pill format for 12 wk experienced decreased visceral fat, TG concentrations, and apolipoprotein B (ApoB) compared with the placebo group [45]. In a second, smaller kochujang trial involving individuals with mild hyperlipidemia, 12 wk of kochujang in pill format resulted in decreased total cholesterol compared with the placebo group and a trend toward comparatively decreased LDL cholesterol concentrations; however, no significant difference was reported for TG concentrations [46]. A 3-arm trial in individuals with overweight or obesity comparing the effects of 12 wk of a pill prepared using traditional kochujang with a high content of beneficial microorganisms (HTK), a pill prepared using traditional kochujang containing a low content of beneficial microorganisms (LTK), and a pill prepared using commercial kochujang, to a total of 25.3 g (19 g/d as kochujang powder) found that only the HTK and LTK groups experienced significant decreases in TC, LDL cholesterol, with visceral fat reduction only in HTK [47]. Although the study did not specify the compositional differences between the low- and high-microbial content preparations, the findings suggest that variations in microbial load and strain diversity (influenced by traditional compared with commercial processing methods) may contribute to the distinct metabolic health effects of kochujang. These improvements in lipid parameters and reductions in visceral fat are particularly relevant to metabolic health, as they may help lower cardiometabolic risk and improve features of MetS, including dyslipidemia and central adiposity. Overall, the evidence for kochujang’s impact on lipid profiles and visceral fat reduction is based on small-to-moderate RCTs with consistent but not large-scale findings.

##### Other botanical fermented products and metabolic health

A crossover study in 21 individuals with prediabetes found that 8 wk of fermented kimchi consumption, compared with nonfermented kimchi, significantly reduced insulin resistance and improved insulin sensitivity, as measured by QUICKI and disposition indices [48]. Improvements in glucose tolerance were observed in 33.3% of participants after consuming fermented kimchi, compared with only 9.5% after the nonfermented intervention. A recent 2024 meta-analysis of intervention studies revealed a significant reduction in fasting blood glucose (WMD: –1.93 mg/dL; 95% CI: –3.82, –0.03; I2 = 17.4%) after the consumption of fermented kimchi compared with control [49]. In another trial, 22 individuals with hypertension and T2DM consumed a traditional Korean diet that included daily servings of kimchi (150 g) and fermented soy condiments (50 g) for 12 wk, alongside regulated quantities of rice, fish, meat, vegetables, and dry-preserved meals [50]. Compared with controls who were instructed to follow standard diabetes dietary guidelines, the intervention group experienced greater reductions in HbA1c and resting heart rate. Although the effects cannot be attributed solely to the fermented components, these findings support a beneficial role for BFFs, such as kimchi, in improving cardiometabolic health. Interestingly, a meta-analysis on vinegar found that consumption was associated with improved fasting blood glucose and HbA1c based on the 5 randomized controlled or crossover trials and 1 quasi-experimental study included [51]. The effect size was greater for the trials that utilized apple cider vinegar [51]. Of the included trials, the apple cider vinegar trial with the lowest bias risk was Kausar et al. [52] which reported improved HbA1c, fasting blood glucose, total cholesterol, TGs, and hip–waist ratio after the 12-wk, 15 mL/d intervention.

Overall, evidence from clinical trials suggests that BFFs such as kimchi and fermented soy condiments, as well as vinegar (particularly apple cider vinegar) can improve insulin sensitivity, glycemic control, and cardiometabolic risk markers, highlighting their potential as complementary dietary strategies for metabolic health.

#### Fermented foods and cardiovascular health

CVD consists of several conditions that may arise related to the heart and blood vessels, which include coronary heart/artery disease, stroke or transient ischemic attack, hypertension, and congestive heart failure, among others. Diet is a recognized lifestyle factor that can shape CVD morbidity and mortality risks [88]. Although dietary advice commonly centers around reductions of salt, red meat, and certain fats alongside increases in whole grains, and other plant foods [88], research indicates that fermented food intake may also play a role in CVD risk management. This is reviewed in the next section.

##### Dairy-fermented foods and cardiovascular health

A 2020 meta-analysis published by Zhang et al. [53] found that, based on 10 cohort studies totaling 385,122 participants, fermented dairy consumption was associated with a decreased risk of general CVD compared with controls (OR: 0.83; 95% CI: 0.76, 0.91), especially with the incidence of CVD (OR: 0.80; 95% CI: 0.72, 0.89). Independently, cheese consumption was associated with decreased risk of CVD (OR: 0.87; 95% CI: 0.80, 0.94), whereas yogurt consumption was associated with even greater protection (OR: 0.78%; 95% CI: 0.67, 0.89 for yogurt) [53]. No relationship was noted between stroke risk and fermented dairy, which aligns with findings from a longitudinal study by Olsson et al. [55] reporting no significant association between fermented dairy consumption and stroke incidence in a cohort of 79,618 Swedish females and males compared with controls. Recent 2025 meta-analysis of 14 prospective cohort studies including 496,631 participants and 24,337 cases reported a statistically significant inverse association between yogurt consumption and CVD risk with a random effect pooled RR of 0.92 (95% CI: 0.87; 0.98, *P* = 0.012) and low heterogeneity (I2 = 19.8%) supporting a potentially protective role of yogurt in CVD prevention [34].

In Savaiano et al.’s [27] systematic review of interventional studies, 16 of the 28 studies analyzing the relationship of fermented dairy products to cardiovascular health and disease found a favorable effect whereas 11 of the remaining studies had neutral outcomes. Multiple high-quality RCTs reported improvements in hypertension or blood lipids with fermented dairy consumption compared with the control groups [27]. In a 2023 meta-analysis, it was found that yogurt improved waist circumference (MD: −3.47 cm; 95% CI: −6.92, −0.02 cm; low certainty), TGs (MD: −0.38 mmol/L; 95% CI: −0.73, −0.03 mmol/L; low certainty), and HDL cholesterol (MD: 0.19 mmol/L; 95% CI: 0.00, 0.38 mmol/L) compared with milk [57].

Evidence for kefir is less consistent. A 2023 systematic review of 18 RCTs reported modest improvements in blood pressure and some improvements in lipid profiles (decreased LDL cholesterol, TG, non-HDL; increased HDL cholesterol), although most trials were judged to have a high risk of bias [37]. By contrast, another 2023 meta-analysis found no significant effects of kefir on lipid profiles [63]. The strongest evidence for lipid improvements appears to come from studies of probiotic fermented milks. Ziaei et al. [56] reported in a meta-analysis of 39 RCTs that probiotic fermented milk consumption significantly reduced LDL cholesterol (WMD: −7.34 mg/dL, 95% CI: −10.04, −4.65, *P <* 0.001) and total cholesterol (WMD: −8.30 mg/dL, 95% CI: −11.42, −5.18, *P <* 0.001), with the most pronounced effects in males. Similarly, RCTs using milk fermented with specific probiotic strains—such as *L. casei* Shirota, *Lactococcus lactis* YIT 2027, and *L. helveticus* LBK-16H—have demonstrated reductions in blood pressure and, in some cases, LDL cholesterol and total cholesterol [58]. For example, Danish males consuming milk fermented with *S. thermophilus* and *Enterococcus faecium* experienced a 10% reduction in LDL cholesterol compared with acidified milk [59]. In another trial, LDL cholesterol and total cholesterol decreased after 4 wk of fermented milk, with maximal reductions at 12 wk but no sustained effects at 6 mo [60]. Additional trials using probiotic yogurt, particularly those enriched with *L. acidophilus* LA5 and *B. animalis* subsp. *lactis* BB12, have reported reductions in LDL cholesterol, total cholesterol, and in some cases blood pressure, TGs, and markers of oxidative stress [36]. Many of these trials employed defined probiotic fermented milk products, suggesting that lipid-lowering and cardiovascular benefits may depend in part on the presence of specific probiotic strains rather than fermentation alone.

Overall, the highest level of evidence on fermented dairy and cardiovascular health remains largely at the biomarker level, where randomized trials and network meta-analyses report modest effects on lipids, blood pressure, and glycemic outcomes. In terms of associative evidence for disease risk, a recent large-scale meta-analysis of prospective cohorts found that total dairy consumption modestly reduced the risk of CVD (∼3.7%) and stroke (∼6%), with cheese and low-fat dairy showing stronger inverse associations [61]. These results are further supported by a meta-analysis of RCTs suggesting that dairy intake may moderately reduce the risk of several health outcomes, extending beyond CVD to include certain cancers [62]. Evidence from a separate meta-analysis examining serving-size effects further strengthens this association: high compared with low yogurt intake was significantly associated with lower risks of all-cause mortality and CVD mortality, whereas each additional daily serving of yogurt was associated with further reductions in CVD mortality (i.e., a 14% lower risk of CVD mortality with each additional serving/d) [64]. For both all-cause and CVD mortality, the benefit plateaued above ∼0.5 serving/d, suggesting a nonlinear dose–response [64]. Although fermented dairy products appear to confer some cardiovascular benefits, results remain variable and may depend on factors such as product serving size and type (e.g., defined probiotic fermented milks compared with general fermented dairy) as well as underlying microbial composition (e.g., as discussed above for varied microorganisms in kefir). Further research should account for these differences when evaluating cardiometabolic effects.

##### Nondairy fermented foods and cardiovascular health

Aside from fermented dairy products, 3 trials involving soy fermentation-based products (either tempeh or kochujang) reported an LDL cholesterol lowering effect [46,47,65]. Another study reported decreased TG concentrations and ApoB associated with a 12-wk kochujang intervention [45]. ApoB is the primary carrier of LDL and chylomicrons through the cardiovascular system, so a decrease in ApoB concentrations may be a protective cardiovascular effect of the intervention [45]. In 2013, a study comparing the effects of low (15 g/d) and high kimchi intake (210 g/d) on serum cholesterol in 100 healthy college-aged individuals found that despite the short 7-d duration, both interventions decreased LDL cholesterol and TC [66]. No significant between-group differences were found. However, the high-dose group did experience a trend toward greater decreases in LDL cholesterol compared with the low-dose group (low: –3.4 ± 10.0 compared with high: –6.9 ± 13.2, *P <* 0.147) [66]. The lack of a control group and the healthy, young population may limit the broader application of these findings although they do point toward a potential benefit of LAB-fermented plant-based foods. Additionally, a trial conducted by Tenore et al. [67] comparing lacto-fermented Annurca apple puree, LAB probiotic alone, and unfermented apple puree in an RCT with 90 individuals found that lacto-fermented Annurca apple puree improved plasma HDL cholesterol and trimethylamine-N-oxide compared with the other groups. All in all, dietary approaches involving fermented foods may largely be aimed at lowering LDL cholesterol and blood TG while raising HDL cholesterol which are the current pharmacologic targets for managing hyperlipidemia in relation to CVD [89]. Once again, the majority of research has centered on the relationship between fermented dairy and CVD risk and has only marginally explored nondairy fermented products.

### Association between fermented foods and cancer

Nutrition has been widely communicated as a factor that shapes risk for various cancers, and which can act as a preventative component of daily lifestyle. Recommendations center around eating a variety of vegetables, fruits, and whole grains and consuming adequate fiber and micronutrients [90]. Considering the potential immunomodulatory, anti-inflammatory, and gut microbiota-modulating effects of fermented foods, they may be a welcome addition for cancer prevention through nutrition [68].

Researchers have proposed that, based on the antioxidant content, microbial presence, and potential to increase short-chain fatty acid producers in the gut microbiome, fermented food consumption may reduce inflammation and even improve response to certain cancer therapies [91]. In vitro investigations indicate that many LAB commonly present in fermented foods have anticancer properties in experimentation [92]. Among these are many probiotic species of *Lactobacillus* and *Bifidobacterium*, which have been reported to improve natural killer cell response, induce tumor cell apoptosis, and inhibit cell proliferation [92]. Safety is a major concern for individuals undergoing cancer therapy due to potentially compromised immune function. Additional data are needed to build knowledge on the safety of fermented foods and LAB in clinical populations.

Findings from Wastyk et al. [68] and Bordoni et al. [28], indicate that fermented foods can alter key inflammatory cytokine concentrations, which may lend insight into the potential protective effects of fermented foods in the context of cancer, which is commonly initiated in association with chronic inflammation and immune dysregulation [93]. However, the health benefit of the observed changes in cytokines in relation to cancer risk protection was not assessed in such studies (results explained in detail later in the review) [70]. Similar analyses and interventional trials to examine effects on inflammation and immune activity would be helpful for understanding the relationship of fermented foods to cancer risk [69].

Although these foods appear to play a beneficial role in supporting human health, association of their intake and risk for specific cancer types shows more variability in current findings. To date, most of the evidence comes from observational cohort, case controls studies. As early as 1989, a case-control study revealed that 225 g or greater daily consumption of fermented milk products resulted in an OR for breast cancer of 0.50 (95% CI) [70]. Zhang et al. [69] conducted a meta-analysis of 61 studies which included 1,962,774 participants and 38,358 cancer cases and found that yogurt intake decreased risk of all cancers (OR: 0.87%, 95% CI: 0.80, 0.95). General fermented dairy intake decreased risk of bladder cancer, colorectal cancer, and esophageal cancer, with stratified analysis revealing a significant protection from colorectal cancer with cheese intake and a significant protection from bladder and esophageal cancers with yogurt intake [69]. A systematic review by Savaiano et al. [27] concluded that the intake of fermented milk was consistently associated with a reduced risk of colorectal and breast cancers. These results are further supported by a meta-analysis of RCTs suggesting that dairy intake may moderately reduce the risk of several health outcomes, extending beyond CVD to include certain cancers [62].

Additional research is needed to clarify the relationship between fermented dairy intake and cancer risk, as well as to establish the underlying mechanisms. Although current evidence suggests a possible association, more clinical studies are required to determine causality. Investigating the mechanistic basis of these potential anticancer effects, whether driven by antioxidants, fiber, or micronutrients present in many BFFs is particularly important. However, the promise of fermented foods for cancer prevention remains insufficiently explored in nondairy fermented foods [94].

### Impact of fermented foods on mental and cognitive health

The gut–brain axis (GBA) refers to the bidirectional communication that links the central and the enteric nervous system [95]. The link between the brain and intestinal function has been implicated in various central nervous system disorders including anxiety, depression, Alzheimer’s disease, Parkinson’s disease, as well as gastrointestinal disorders such as irritable bowel syndrome (IBS) [[95], [96], [97]]. Altered gut microbial profiles have been observed in populations with these disorders compared with healthy controls, suggesting a relationship between the gut microbiota and these disease states, which may exist through the GBA [[95], [96], [97], [98]]. Researchers propose that the mechanism of this relationship may involve the ability of the gut microbiota to directly interact with neurons through neuron–bacteria signaling [71], as well as indirectly influencing neuronal function by modulating local and systemic inflammation, regulating immune responses, producing bioactive metabolites, and contributing to neurotransmitter synthesis and activity [97,99]. With this in mind, it follows that trials investigating the impact of dietary patterns that may beneficially modulate the gut microbiota, such as diets high in fiber and/or fermented foods, are of interest. Observational and interventional studies have begun interrogating the relationship between fermented foods and neuropsychological symptoms, including stress, depression, and anxiety-related symptoms, among others. The following section reviews the evidence in this area.

#### Fermented foods and mood disorders

A meta-analysis of 8 prospective cohort studies (83,533 participants) found that fermented dairy intake, including cheese and yogurt, was associated with a lower risk of depression. In a trial combining fermented foods with a prebiotic-rich diet, 24 healthy adults showed greater reductions in perceived stress over 4 wk, alongside changes in fecal lipids and urinary tryptophan metabolites [54]. However, as the intervention included both fermented and fiber-rich foods, the effects cannot be attributed to fermented foods alone. In another study, 479 female healthcare workers consuming yogurt fermented with *L. delbrueckii* ssp. *bulgaricus* OLL1073R for 16 wk reported improvements in sleep and gastrointestinal quality of life compared with controls [72]. Live microbes in fermented foods may reduce dysbiosis and improve gut microbiota, potentially mitigating neuropsychiatric symptoms, with certain strains (e.g., *S. thermophilus*, *L. plantarum*, and *B. adolescentis*) linked to increased gamma-aminobutyric acid bioavailability [100]. Hilimire et al. [73] reported an inverse association between fermented food intake and social anxiety in young adults.

Evidence remains limited, with few interventional studies and mixed observational findings. For instance, a study of 372 medical students under examination stress found higher fermented food intake associated with increased anxiety and depressive symptoms [101], whereas Kinoshita et al. [72] observed improved psychological quality of life during a 16-wk yogurt intervention. Karbownik et al. [74] found divergent effects depending on baseline psychological health.

#### Fermented foods and cognition

Building on the GBA paradigm, researchers have begun exploring the potential of fermented foods to enhance cognition and protect against age-related cognitive decline. Marx et al. [102] conducted a meta-analysis of existing RCTs examining fermented food interventions and cognitive outcomes. Of the 6 included studies, 4 focused on milk fermented with probiotic strains. Although trends suggested possible benefits, no significant effects were observed on global cognition or specific cognitive domains. A separate subgroup analysis of prebiotics and probiotics also yielded nonsignificant effects, with even weaker trends. The authors highlighted the limited number of studies, small sample sizes, heterogeneity of interventions, and variability in outcome measures as likely factors preventing the detection of significant effects. These findings underscore the need for more rigorous interventional trials across diverse populations and fermented food types.

Evidence also suggests a potential protective role of fermented foods against age-related neurologic diseases such as dementia and Alzheimer’s disease. A recent systematic review of 22 cohort, 4 case-control, and 3 cross-sectional studies concluded that consumption of fermented foods and beverages (such as coffee, soy products, and high-fermented-food diets) was associated with a lower risk of Alzheimer’s disease and dementia [103]. Supporting this, an RCT with 30 participants with Alzheimer’s disease found that daily consumption of 200 mL milk fermented with *L. acidophilus*, *Lacticaseibacillus casei*, *B. bifidum*, and *Limosilactobacillus fermentum* (2 × 10^9^ CFU/g each) for 12 wk improved mini-mental state examination scores compared with controls [104]. These findings suggest that dietary patterns including multiple fermented food items, such as a Mediterranean-style diet, may confer neuroprotective benefits. Indeed, systematic reviews indicate that adherence to Mediterranean diets is associated with reduced risk of Alzheimer’s and Parkinson’s disease, potentially mediated through effects on gut microbial composition [105,106]. Although these diets contain a variety of nutritionally dense and polyphenol-rich foods, the specific contributions of fermented foods warrant further investigation to clarify their role in cognitive health and aging.

In summary, emerging evidence suggests that fermented foods may influence neuropsychological health through modulation of the GBA, largely via effects on gut microbiota composition, immune function, and inflammation. Observational and interventional studies show promising associations with stress, mood, and cognitive function, but findings are inconsistent, likely due to differences in individual health, stress levels, types of fermented foods, and study design. Although some studies link fermented food intake to reduced depressive and anxiety symptoms, others report potential negative effects under certain conditions. Meta-analyses on cognition in aging populations show limited significance, although specific foods and dietary patterns, such as the Mediterranean diet, may help mitigate cognitive decline. Overall, the research highlights growing interest in fermented foods for mental and cognitive health, but well-designed, large-scale trials are needed to confirm efficacy.

### Functional attributes of fermented foods that might support human health

**FIGURE 3:**
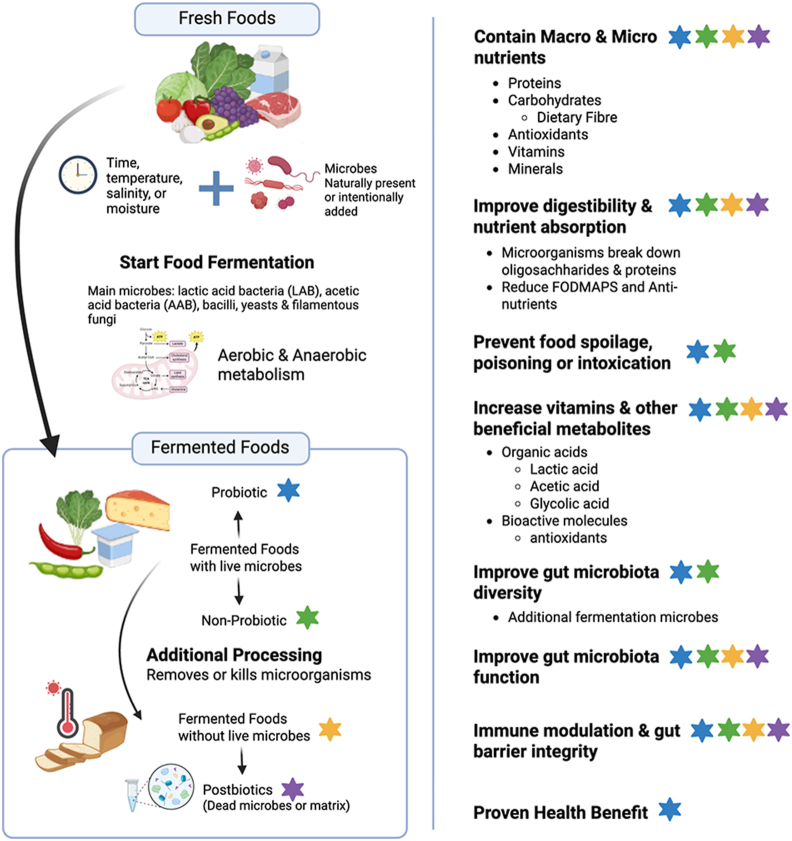
Fermented foods improve digestibility, nutrient bioavailability, and gastrointestinal health. Fermentation processes offer a promising approach to enhance food nutrient content and bioavailability. Through the production of beneficial microbial metabolites, fermented foods can support human health and reduce food spoilage. Additionally, the presence of live microorganisms in these foods promotes nutrient digestibility, enriches gut microbiota diversity, and modulates the immune system. Stars are placed to indicate which benefits are associated with each type of fermented food. FODMAPs, fermentable oligosaccharides, disaccharides, monosaccharides and polyols.

Health benefits of fermented foods might arise from their overall characteristics, including the nutrients in the starting material, molecular changes during fermentation, microbial metabolites, and any remaining microbes (Figure 3). Fermentation can enhance nutritive qualities by breaking down oligosaccharides or proteins, synthesizing vitamins, and reducing antinutrients such as tannins and phytic acid, improving nutrient bioavailability [2,107,108]. For example, LAB-fermented cow and soy milk curds show increased protein availability, folate content rises in *Saccharomyces cerevisiae*-fermented rye flour, and iron availability improves in LAB-fermented vegetables [109,110]. Strategic fermentation can thus enhance nutritional value. Fermented foods also contribute essential nutrients and prebiotic fibers that support health. Kimchi increases dietary fiber intake [111], and both pasteurized and nonpasteurized sauerkraut improved IBS symptoms and altered gut microbiota, highlighting benefits from prebiotic fibers independent of live microbes [112]. Yogurt and cheese are rich sources of calcium and vitamin D. Cheese often contains more vitamin D than milk due to its higher fat content, whereas yogurt can provide more calcium than milk. These examples illustrate that many fermented foods support health not only through their microbial or fermentation-related properties but also via their macro- and micronutrient content. Such nutritional enhancements likely contribute to the beneficial health effects associated with fermented food consumption.

Food fermentation produces bioactive molecules such as phenols, peptides, and organic acids, which depend on the starting material and microbes involved [1,113,114]. Bioactives can exert physiological effects beyond classical nutrition and are often explored for pharmaceutical and industrial applications. For example, soy isoflavones can be transformed during fermentation into more bioactive forms with high antioxidant activity [115]. These compounds may protect against oxidative stress and inflammation-related conditions. Further modification of these bioactives by gut microbiota may contribute to the health effects of fermented foods, but additional mechanistic and human studies are needed. Understanding the potential for bioactive molecules in fermented foods to support human health requires further investigation. In parallel, growing attention is being given to the role of the fermented food matrix itself, which may influence the stability, release, and activity of bioactive compounds. This interest has expanded into the field of postbiotics, defined by international consensus as “preparations of nonviable microorganisms and/or their components that confer health benefits to the host.” These microbial products and metabolites may act independently of live microorganisms, offering new avenues to explain the health-promoting effects associated with fermented foods.

Fermented foods that contain live cultures can introduce a dose of microbes to the human gastrointestinal system [2]. The quantity of microbes received via food will vary greatly based on food choices, as demonstrated by Lang et al. [116], who found that an American convenience food diet contributed 1.4 × 10^6^ CFU/d, a vegan diet contributed 6 × 10^6^ CFU/d, and the USDA-recommended diet contributed 1.3 × 10^9^ CFU/d. These microbes may indirectly alter the commensal gut microbial ecology in beneficial ways, allowing for increased diversity and proliferation of health-associated members. Studies on the survival of fermented food microbes as they pass through the human gastrointestinal tract have found that the microbes can survive in appreciable quantities and affect the gut microbiota in both the short and long term as reviewed by Leeuwendaal et al. [117]. Although it is not clear that eating particular foods will result in defined gut microbial changes, ≥16 trials have reported significant alterations in gut microbiota after fermented food interventions [118]. Some of these included increases in microbes directly involved in fermentation, such as *Leuconostoc mesenteroides* from camembert cheese and *Lactobacillus* from fermented milk [118]. Others reported shifts in commensal taxa, such as decreases in *Clostridia* abundance after fermented soybean milk consumption [118]. Two more recent studies have contributed to this body of evidence and are further discussed in the immune function section. Wastyk et al. [68] found that several taxa, including *Roseburia* and *Lachnospiraceae*, correlated with fermented food intake, likely reflecting indirect effects on the existing microbiota rather than direct microbial introduction. Similarly, van den Belt et al. [75] reported that a high-fermented-food intervention increased *Anaerostipes*, *Faecalibacterium*, and *Bifidobacterium* spp. Notably, a consensus on what constitutes a “healthy” or “beneficial” gut microbiome does not currently exist. Therefore, interpreting changes in microbial community compositions as favorable requires cautious consideration. Reported microbial alterations in some studies coincided with improvements in metabolic markers, although the clinical relevance of these markers remains uncertain.

### Effects of fermented foods on digestibility, nutrient bioavailability, and gastrointestinal function

Another potential role of fermented foods in improving health status and nourishment arises from increases in food digestibility and decreases in antinutritive factors following certain fermentation processes (Figure 3) [2]. This may be key for susceptible populations such as individuals with IBS who are sensitive to fermentable oligosaccharides, disaccharides, monosaccharides, and polyols (FODMAPs), individuals with lactose malabsorption, or individuals who struggle to meet iron needs, as well as the general public [2]. Lactose intolerance, caused by reduced lactase enzyme activity, is common worldwide and influenced by genetic, dietary, and cultural factors [67]. Fermented dairy products, which contain reduced lactose as a result of microbial fermentation, offer a more tolerable alternative. This microbial fermentation mimics the action of the lactase enzyme, breaking down lactose into glucose and galactose, thereby facilitating absorption. Because milk is recommended for key nutrients such as calcium, fermented dairy foods like cheese and yogurt can provide these nutrients without causing discomfort. Cheese fermentation reduces lactose, and residual lactose is largely removed in the whey component of the cheese-making process [2]. In yogurt, LAB deliver microbial lactase enzymes that remain active in the human digestive tract, where they help break down residual lactose into glucose and galactose. This enhances lactose digestion (via enzymatic hydrolysis) and facilitates the subsequent absorption of these monosaccharides [119]. Yogurt has been found to be better tolerated than milk treated with commercial lactase [120]. A systematic review by Savaiano et al. [27] concluded that yogurt causally improves lactose digestion and tolerance, based on 7 high-quality RCTs in participants aged 7 mo to 53 y. Kolars et al. [121] detected microbial beta-galactosidase in the intestines, and Martini et al. [122] showed that only *S. thermophilus* and *L. delbrueckii subsp. bulgaricus* fermentation significantly improved lactose tolerance. Importantly, pasteurization eliminates this benefit, suggesting that when live microbes are present, they continue to act during transit through the gastrointestinal tract, acting on lactose in the gut lumen rather than solely during the fermentation process.

Fermentation of vegetables and grains can also improve bioavailability of micronutrients like iron [109,123,124]. Iron is required for oxygen transport, energy production, and DNA synthesis. Lactic acid fermentation increases the bioavailability of iron from food [124]. Researchers investigated iron absorption in 17 participants who consumed wheat flour and wheat bran rolls with either *L. plantarum* fermented vegetables or unfermented vegetables over 4 d and found that after 2 wk, the group consuming the fermented vegetables had higher blood iron content [124]. The researchers attribute this to a greater ratio of available Fe^3+^ to Fe^2+^, which appears to be favorable for absorption as demonstrated by their in vitro experiments with human intestinal cells [124]. In other foods, lactic acid fermentation may decrease antinutritive phytate content of the foods, increasing nutrient availability. Phytate is found in several seeds, grains, and legumes and its negative charge can result in it bonding to positively charged iron molecules from food, preventing their absorption in the gastrointestinal tract [123]. Lactic acid fermentation can decrease phytate content and increase iron solubility from cereals and grains including whole wheat, oats, sorghum, and maize, increasing the nutritive value of these foods [109,123]. Fermentative processes may also increase the availability of other minerals such as calcium, magnesium, and zinc, and improve protein digestibility from grains [110]. As such, including LAB-fermented foods such as sourdough wheat bread or fermented vegetables may help support iron and other micronutrient concentrations, especially in individuals who are at risk for deficiency or who adhere to a more plant-based dietary pattern in which bioavailability might be a challenge.

Individuals with IBS can experience symptom aggravation from consuming FODMAPs found in common foods such as milk, garlic, beans, and rye among many others [125]. For these individuals, reducing dietary FODMAP load can reduce symptoms like bloating, gas, and diarrhea [125]. Sourdough wheat bread fermentation has been demonstrated to reduce FODMAP content of bread compared with fermentation with *S. cerevisiae*, reducing fructans by nearly 75% [126]. In an RCT by Laatikainen et al. [127], 4-wk of consuming a low-FODMAP rye sourdough fermented bread resulted in lower flatulence, abdominal pain, cramps, stomach rumbling, and breath hydrogen concentrations compared with the regular yeast fermented rye bread control group. Thus, for individuals with sensitivity to FODMAPs, choosing LAB-fermented products may improve tolerability and facilitate the nutritional and/or experiential benefits of consuming foods that may otherwise cause discomfort.

### Effects of fermented foods on immune function

Immune system function is paramount for maintaining overall health and preventing disease or infection. From birth, the human immune system is shaped by nutritional and microbial inputs, and this interwoven relationship between food, gut microbiota, and immunity persists throughout life [128,129]. Fermented foods can contribute to this relationship by providing nutrients that may influence immune health and, in the case of fermented foods containing live cultures, by introducing microorganisms to the gut microbiota [68]. Few interventional trials have assessed the impact of fermented foods on immune health. Several markers have been used to assess immune function in humans, but many are criticized for their lack of specificity and reproducibility. Circulating cytokines (e.g., IL-6, TNF) and CRP are widely measured yet strongly influenced by external factors such as stress, infection, and obesity, limiting their interpretability. Functional assays, including lymphocyte proliferation and natural killer cell activity, are also variable and poorly standardized. Even commonly used endpoints like secretory IgA or vaccine response provide only a partial view of immune competence. Together, these limitations highlight the ongoing challenge of identifying reliable, clinically relevant markers of immune dysfunction in human studies that could be used in fermented food interventions.

Wastyk et al. [68] compared 10-wk high-fiber and high-fermented food diets in 39 healthy adults. Fiber intake increased from 21.5 ± 8.0 to 45.1 ± 10.7 g/d, raising CAZyme activity but causing minimal changes in microbiota composition or inflammatory cytokines. In contrast, fermented food intake rose from 0.4 ± 0.6 to 6.3 ± 2.9 servings/d, increasing gut microbial diversity and reducing cytokine markers including IL-6, IL-10, and IL-12ß. Building on this, van den Belt et al. [75] examined 147 adults in an 8-wk intervention. The high-fiber group (+10.3 g/1000 kcal/d) enhanced butyrogenic potential, reduced transit time, and improved sleep, whereas the high-fermented food group (+6.3 servings/d) increased immune markers including CD5, CD6, CD8A, IL-18R1, and SIRT2 [75]. These studies illustrate that fermented foods and fiber modulate the gut microbiome and immune system through distinct pathways. Although mechanistic biomarkers such as CAZymes and shifts in taxa highlight diet–microbiome interactions, functional outcomes such as transit time and sleep are closer to health measures. Overall, diets rich in fermented dairy and vegetables may reduce inflammatory cytokines and alter the gut microbiome, although the clinical relevance remains uncertain.

During development, early interactions between the host immune system and the microbiota have profound and long-term implications for human health [130]. Associative evidence from a 2022 birth cohort study aligns with prior preclinical evidence to indicate that the maternal gut microbiota composition observed during pregnancy is associated with infants’ subsequent innate and adaptive immune characteristics after birth [131]. In another study, daily maternal consumption of yogurt, cheese, and fermented olives during pregnancy was found to be higher in the control group compared with the atopic dermatitis group, and the diversity of fermented foods consumed was also associated with reduced risk of atopic dermatitis (OR: 0.27; 95% CI: 0.14, 0.53) [76]. Further support for a *causal* relationship between fermented food consumption and immune function comes from clinical trials and meta-analyses of intervention studies. He et al. [77] systematically reviewed the literature to explore this relationship through 2 case-control studies and 8 clinical trials involving females at various stages of pregnancy. Probiotic yogurt consumption was associated with beneficial immune-system-related outcomes for the mother, including decreased risk for gestational diabetes, reduced inflammatory response as measured by glutathione reductase and high-sensitivity CRP concentrations, and successful treatment of bacterial vaginosis [77]. Similarly, Rashidi et al. [78] meta-analysis of 22 RCTs revealed a decreased risk of pneumonia (RR: 0.76; 95% CI: 0.61, 0.95) and common cold (RR: 0.68; 95% CI: 0.49, 0.96) with probiotic fermented dairy food consumption across child and adult populations compared with placebo.

Overall, current evidence suggests that fermented foods can beneficially influence immune function and lower infection risk, but most findings are limited to probiotic-containing products. Early evidence also suggests that these benefits may apply during pregnancy to support maternal and child health at a crucial developmental stage. More research is needed to determine whether these effects extend to nonprobiotic fermented foods and to identify robust, clinically relevant immune markers to clarify mechanisms. If confirmed, fermented foods could represent an accessible, cost-effective dietary strategy for supporting immune health at the population level.

## Safety and Other Considerations

The diverse microbiological activity that occurs during fermentative processing and continues in many final products contributes to the potential health benefits of the foods [2]. However, to achieve the desired product, conditions of the fermentation including temperature, time, substrate(s), pH, and moisture must fall within specific ranges to prevent growth of undesirable bacteria, yeasts, or fungi that could be pathogenic or toxic. In the commercial setting, the risk of unintended microbiological contamination or growth can be mitigated using Hazard Analysis and Critical Control Points, standard operating procedures, excellent food safety and manufacturing training for involved staff members, and product monitoring. Fermentation in the at-home setting can present additional challenges. Because there may not be rigorous quality control over ingredients that are used in fermentation, processing steps, or storage conditions during the fermentation, there is greater risk of unknown contamination, unintended microbial growth, and difficulty determining the safety of consumption due to lack of testing [132]. Therefore, the safety of home-fermented foods depends largely on an individual’s ability to follow reliable guidance. To ensure safe practices, individuals should learn from reputable resources or knowledgeable experts, which can help them gain the skills and confidence needed to produce their desired food and recognize when something has gone wrong. Adhering to appropriate sources helps prevent contamination and ensures proper fermentation conditions, ultimately leading to safe and beneficial outcomes.

Despite the general safety of commercially available fermented foods, data on potential side effects remain limited. In a systematic review of kefir, Kairey et al. [37] noted that only a fraction of the trials included adverse event monitoring and reporting [37]. Among those that did, some gastrointestinal symptoms and discomfort were observed, particularly in potentially susceptible populations such as individuals undergoing chemotherapy. This example illustrates how the measurement and reporting of side effects in fermented food clinical trials is often inconsistent. Moreover, as highlighted by Caffrey et al. [133], although fermented foods may confer health benefits for many, certain populations should exercise caution, including immune-compromised individuals, pregnant females, and those with histamine intolerance, IBS, or small intestinal bacterial overgrowth. This underscores the importance of establishing well-tolerated intake ranges through careful adverse event monitoring and reporting, especially in populations beyond generally healthy adults.

## Research Gaps and Future Directions

**FIGURE 4:**
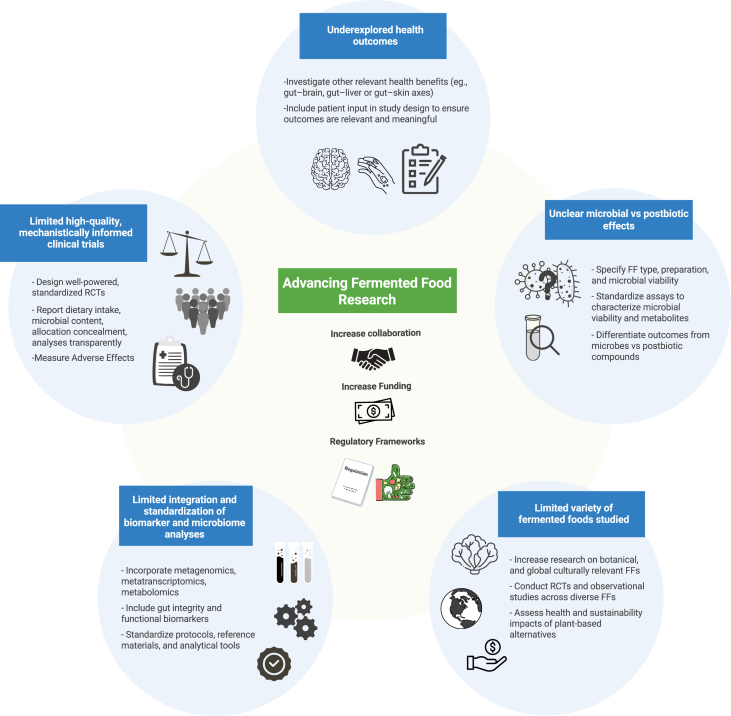
Key research gaps and proposed solutions in fermented foods science. There are major research gaps in fermented food science, including limited clinical trials, understudied populations, narrow food type focus, unclear mechanisms, microbial characterization challenges, and incomplete nutritional data. Addressing these areas through targeted research and standardized methodologies, increased collaboration and funding will support the development of evidence-based dietary recommendations for fermented foods and human health. FF, fermented foods; RCT, randomized controlled trial.

Despite promising evidence linking fermented foods to improved health outcomes, important limitations remain. Much of the literature is observational, limiting causal inference and often confounded by broader dietary and lifestyle factors. Interventional studies, while encouraging, are generally small, short-term, and heterogeneous in terms of fermented food types and comparators. Variability in microbial composition between traditional and commercial products further complicates interpretation, as strain diversity and viability likely influence outcomes. Standardized methods for quantifying live microbes are emerging, but mechanistic studies are still limited in clarifying whether benefits are driven by the food matrix, microbial activity, or both. Practical challenges also exist. Reviewing the available evidence reveals substantial variability in reported dosages across studies. This underscores the lack of a standardized “effective dose” for fermented foods, with intake levels differing according to food type, fermentation method, and study design. Moreover, most systematic reviews do not specify a clear suggested or average intake, leaving dosage interpretation dependent on individual trial data. Additional evidence is therefore needed to establish broader consensus dosage recommendations. Compounding this challenge, many commercial products contain too few microbes to deliver effects at typical consumption levels, emphasizing the need for improved formulations and standardized microbial quantification. One proposed solution, suggested by Marco et al. [134], is to classify foods by low, medium, or high live microbial content, thereby providing benchmarks to guide product development, labeling, and formulation. Overall, significant gaps remain in the evidence base for fermented foods. The following section explores these gaps in greater depth, with a summary presented in Figure 4.

### Limited high-quality, mechanistically informed clinical trials

Although observational studies are integral to understanding the relationship between food intake levels and disease incidence, additional well-designed RCTs have been called for by several key systematic reviews to explore potential causal links between fermented foods and various health impacts [37,42,113,135]. These trials would also help establish intake range recommendations and safety knowledge. In doing so, researchers urge improving the quality of research through standardized trial designs that properly capture dietary intake, use of reference standard methods, analysis of the microbial content of the fermented food tested, and transparent reporting of allocation concealment, analyses, and findings [87,113]. Collaborative efforts among research institutions, industry partners, and regulatory bodies are critical to promoting best practices and ensuring robust, interpretable data. Standardization initiatives are fundamental for advancing our understanding of the microbiome’s role in health and disease and for translating research findings into meaningful clinical applications.

### Limited integration and standardization of biomarker and microbiome analyses

Relatively few studies have incorporated gut microbiome analyses into their interventional designs, which is understandable given the primary aim of many trials is to assess health outcomes rather than mechanisms, and the high costs associated with such analyses. Nonetheless, when the objective is to explore the role of the gut microbiome in mediating health effects, the inclusion of methods such as metagenomics, metatranscriptomics, and microbial metabolomics can provide valuable mechanistic insight and strengthen the interpretation of findings. This may yield some mechanistic insight into any observed health impacts. This is particularly relevant for foods containing live cultures, but also important for those that do not but still contribute prebiotic fibers, phenols, bioactive molecules, and other microbial metabolites which are components that may still modulate the microbiota despite the absence of live microbes. Therefore, whenever possible, incorporating measures of gut integrity, functional biomarkers, and “omics” can help elucidate the mechanisms underlying observed effects and align them with clinical outcomes. This, in turn, can support the validation of candidate biomarkers as clinical endpoints in future trials, ultimately strengthening the translational value of short-term human studies, where direct health outcomes may be difficult to capture but biomarker improvements can serve as early indicators of potential health benefits.

Challenges remain in standardizing microbiome research, including variability in sample collection, DNA extraction, sequencing, and bioinformatic analyses, which can bias microbial profiles. The lack of standardized reference materials and analytical tools limits reproducibility and comparability across studies [136]. To address this, initiatives such as the National Institute of Standards and Technology in the United States, the Institute for Reference Materials and Measurements in Belgium, and the European Committee for Standardization are developing reference materials, validated protocols, and preanalytical standards to harmonize microbiome research across laboratories. Standardizing methodologies, specifying the types of fermented foods administered, and harmonizing analytical approaches are crucial to advance mechanistic understanding.

### Underexplored health outcomes

Most of the current evidence on fermented foods has focused on cardiometabolic health outcomes and immune modulation. However, as research into the gut and its microbial communities continues to evolve, it is becoming increasingly evident that these communities influence multiple physiological axes, including the gut–brain, gut–liver, and gut–skin axes. Fermented foods may modulate these pathways by affecting neurotransmitter production, stress responses, liver function, and systemic inflammation, highlighting their potential role in neurocognitive, emotional, liver, and skin health. Exploring the effects of fermented foods on these broader health domains represents an important opportunity for future research. Trials incorporating relevant biomarkers and mechanistic endpoints could provide insight into how these foods influence physiology beyond cardiometabolic and immune parameters.

### Unclear mechanisms: microbial compared with postbiotic effects

A key gap in fermented food research is disentangling whether health effects arise from the activity of live microorganisms or from nonviable microbial components and metabolites (parabiotic and postbiotic effects). Many fermented foods undergo pasteurization or processing steps that inactivate microbes, yet they may still confer nutritional or physiological benefits through bioactive compounds, peptides, or altered nutrient profiles. Conversely, foods containing live cultures may influence gut microbiota composition and function in ways not achievable with inactivated products. To better discern these effects, fermented food-based interventions should clearly specify the type of fermented foods consumed, including preparation methods and microbial viability. Clarifying these mechanisms is essential to guide both research and dietary recommendations. Future studies should explicitly distinguish between microbial and postbiotic effects, using standardized methods to characterize microbial viability, metabolic products, and their respective contributions to health outcomes.

### Limited variety of fermented foods studied

Although fermented dairy foods have received the most research attention, there are clear opportunities to expand knowledge to other products. To date, the literature remains heavily biased toward Western dairy fermentation, with most systematic reviews focused on cheese and yogurt, reflecting both the larger evidence base and the influence of industry size and funding. In contrast, research on BFFs such as kimchi, kombucha, and tempeh remains limited. Expanding studies to globally consumed fermented foods (e.g., natto, doubanjiang, fermented fish sauce, koji) would help broaden understanding of their health impacts across diverse populations. Because plant-based meat and dairy alternatives continue to grow in the market, it is critical to evaluate whether these fermented products confer benefits comparable to dairy-fermented products. Such work is important not only for functional food development and consumer adoption but also for reducing reliance on animal-based products to meet population needs and sustainability goals [137]. Increasing research on botanical and culturally significant fermented foods is also relevant from a societal perspective, helping to preserve traditional practices (e.g., Indigenous foods) while exploring how cultural and genetic contexts may shape health benefits [138]. Finally, as consumer demand rises, systematic investigation of plant-based and novel fermented products will be essential to clarify their potential health effects and guide innovation in the fermented plant-based food sector.

### Need for standardized monitoring of adverse effects

Many studies lack standardized protocols for adverse event monitoring, which can lead to underreporting and hinder accurate assessment of safety [37]. Consequently, fermented foods are sometimes perceived as universally safe, despite the possibility of adverse effects in certain groups. To address this gap, future research should prioritize rigorous, standardized methods for monitoring and reporting adverse events, providing clearer insights into the safety profile of fermented foods. Such evidence would support the development of population-specific, evidence-based recommendations, allowing the benefits and potential risks of fermented food consumption to be appropriately balanced.

### Need for regulatory frameworks

Fermented foods are widely consumed for cultural, culinary, and nutritional reasons. Integrating them into national dietary guidance would enhance consumer awareness and support future research, paving the way for more targeted health recommendations. A practical first step would be to recognize fermented foods as a distinct dietary category and include them in Food Guides as examples across existing groups such as dairy, vegetables, legumes, and grains. A review of nutritional guidelines worldwide showed that only India’s guide explicitly encourages the consumption of fermented foods [139]. However, this appears to be the exception, as most countries do not. This foundational framing could be complemented with clear explanations of what fermented foods are, why they are widely consumed, and their cultural and nutritional significance. Educational messaging could then emphasize their contributions to nutrient intake, digestibility, and dietary diversity, while avoiding specific health claims until stronger interventional evidence becomes available. Interim guidance could encourage the selection of minimally processed options with lower added sugar and sodium and clarify the distinction between foods containing live cultures and those that do not. Formally signaling fermented foods within national guidelines would not only promote consumer awareness but also stimulate further research, laying the groundwork for more specific and evidence-based recommendations as the scientific evidence grows. As robust interventional evidence accumulates, fermented foods could be promoted for both cultural and health benefits, enabling clinicians to recommend them confidently. Broader acceptance by healthcare professionals will require formal endorsement from regulatory bodies, supported by large-scale RCTs with standardized definitions, microbial reporting, and clinically relevant endpoints, as well as systematic assessments of long-term safety, dose–response relationships, and population-specific effects.

Fermented foods hold potential to modulate the gut microbiota and support diet-based strategies for promoting gut health. Observational evidence links their consumption to reduced risk or improved management of chronic conditions, although more interventional studies are needed to establish causality. Stronger evidence will be critical to shape dietary recommendations that differentiate between healthy populations, where fermented foods may serve a preventive role, and clinical populations, where they could complement treatment. Ultimately, clear, evidence-based guidance tailored to health status, intake frequency, and food type will be essential to translate research into practice.

## Author contributions

The authors’ responsibilities were as follows – BPW, RAR, JPB: designed research; KS: conducted research; KS, CSM, ST, YF: analyzed data and wrote the paper; CLS-L: wrote the paper; and all authors: read and approved the final manuscript.

## Data availability

Data described in the manuscript, codebook, and analytic code will be made available upon request pending application and approval.

## Funding

This work was supported by a Weston Family Foundation grant for the Canadian Fermented Foods Initiative – Phase II, administered through Lawson Research Institute.

## Conflict of interest

JPB reports financial support was provided by Weston Family Foundation. All other authors report no conflicts of interest. Figure graphics were created with BioRender (www.biorender.com) under Jeremy Burton’s Premium Account.
